# Perioperative Right Ventricular Dysfunction and Abnormalities of the Tricuspid Valve Apparatus in Patients Undergoing Cardiac Surgery

**DOI:** 10.3390/jcm12227152

**Published:** 2023-11-17

**Authors:** Alessia Mattei, Alessandro Strumia, Maria Benedetto, Antonio Nenna, Lorenzo Schiavoni, Raffaele Barbato, Ciro Mastroianni, Omar Giacinto, Mario Lusini, Massimo Chello, Massimiliano Carassiti

**Affiliations:** 1 Anesthesia and Intensive Care Operative Unit, Fondazione Policlinico Universitario Campus Bio-Medico, Via Alvaro del Portillo 200, 00128 Rome, Italy; a.mattei@policlinicocampus.it (A.M.); a.strumia@policlinicocampus.it (A.S.); l.schiavoni@policlinicocampus.it (L.S.);; 2 Cardio-Thoracic and Vascular Anesthesia and Intesive Care Unit, IRCCS Azienda Ospedaliero-Universitaria di Bologna, Via Albertoni 15, 40123 Bologna, Italy; maria.benedetto@aosp.bo.it; 3 Cardiac Surgery Unit, Fondazione Policlinico Universitario Campus Bio-Medico, Via Alvaro del Portillo 200, 00128 Rome, Italy; 4 Anesthesia and Intensive Care Research Unit, Fondazione Policlinico Universitario Campus Bio-Medico, Via Alvaro del Portillo 200, 00128 Rome, Italy

**Keywords:** right ventricle, tricuspid valve, cardiac surgery, right ventricular, dysfunction

## Abstract

Right ventricular (RV) dysfunction frequently occurs after cardiac surgery and is linked to adverse postoperative outcomes, including mortality, reintubation, stroke, and prolonged ICU stays. While various criteria using echocardiography and hemodynamic parameters have been proposed, a consensus remains elusive. Distinctive RV anatomical features include its thin wall, which presents a triangular shape in a lateral view and a crescent shape in a cross-sectional view. Principal causes of RV dysfunction after cardiac surgery encompass ischemic reperfusion injury, prolonged ischemic time, choice of cardioplegia and its administration, cardiopulmonary bypass weaning characteristics, and preoperative risk factors. Post-left ventricular assist device (LVAD) implantation RV dysfunction is common but often transient, with a favorable prognosis upon resolution. There is an ongoing debate regarding the benefits of concomitant surgical repair of the RV in the presence of regurgitation. According to the literature, the gold standard techniques for assessing RV function are cardiac magnetic resonance imaging and hemodynamic assessment using thermodilution. Echocardiography is widely favored for perioperative RV function evaluation due to its accessibility, reproducibility, non-invasiveness, and cost-effectiveness. Although other techniques exist for RV function assessment, they are less common in clinical practice. Clinical management strategies focus on early detection and include intravenous drugs (inotropes and vasodilators), inhalation drugs (pulmonary vasodilators), ventilator strategies, volume management, and mechanical support. Bridging research gaps in this field is crucial to improving clinical outcomes associated with RV dysfunction in the near future.

## 1. Introduction

Perioperative right ventricular (RV) dysfunction and tricuspid valve abnormalities in cardiac surgery remain intricate components in the orchestration of circulatory function, continuously challenging the anesthesiologist, the cardiac surgeon, and all the perioperative care team. The right ventricle, often overshadowed by its left counterpart, is crucial for maintaining a hemodynamic equilibrium and needs to adapt and respond to the hemodynamic alterations caused by surgery. Moreover, the coexistence of tricuspid valve pathologies with RV abnormalities exacerbate the challenges in achieving favorable surgical outcomes. This narrative review aims to understand the role of perioperative RV dysfunction and tricuspid valve anomalies, analyzing mechanisms, clinical implications, diagnostic tools, and strategies for its effective management to finally improve patients’ surgical recovery and long-term prognosis.

## 2. The Clinical Problem: A Matter of “Definition”

RV dysfunction of any grade is a common occurrence following cardiac surgery, and it is becoming clinically significant in 2.9% of cardiac surgical cases [1]. However, the incidence of RV failure after elective cardiac surgery is relatively low, occurring in approximately 0.1% of patients. On the other hand, in the context of heart transplant and left ventricular assist device (LVAD) implantation, the incidence significantly rises, reaching peak rates of 30–40% [2,3,4,5,6]. RV dysfunction has important clinical consequences; a study conducted by Bootsma et al. involving 1109 cardiac patients demonstrated an independent association between RV dysfunction and 2-year all-cause mortality after heart surgery [7]. Levy et al., in their study, further revealed that RV dysfunction was associated with significant adverse outcomes post-cardiac surgery, including death, reintubation, strokes, and prolonged ICU stays [1].

In the perioperative setting of cardiac surgery, a universally accepted definition of RV dysfunction remains elusive, despite various criteria proposed by authors based on echocardiography and hemodynamic parameters [8]. In their recent study on postoperative RV dysfunction after cardiac surgery, Levy et al. identified postoperative RV dysfunction as a combination of hemodynamic instability requiring both inotropic/vasopressor drugs and pulmonary vasodilators, accompanied by echographic signs of right heart dysfunction [1]. More precisely, Jabagi et al. proponed an accurate definition of perioperative acute RV dysfunction [9]. According to the authors, intraoperative failure should comprehend the following:

- Difficult weaning from a cardiopulmonary bypass (CPB), accompanied by an administration of >1 vasopressor and >1 inotropic drug/inhaled pulmonary vasodilator OR >1 weaning from a CPB attempt (assuming RV dysfunction to be the cause) OR weaning from a CPB only obtained through mechanical support.
- Direct visualization of RV dysfunction or a >20% reduction in RV fractional area measured with echocardiography.

Postoperative failure signs should comprehend the following:

- Central venous pressure (CVP) > 15 mmHg or the cardiac index (CI) < 1.8 L/min/m^2^.
- Absence of elevated left atrial pressure (LAP)/pulmonary capillary wedge pressure (PCWP) > 18 mmHg/tamponade/ventricular tachycardia/pneumothorax.
- RV stroke work index (RVSWI) < 4, where RVSWI = 0.136 × stroke volume index (SVI) × (mean pulmonary arterial pressure (mPAP) − right atrial pressure (RAP)) and SVI = stroke volume × body surface area (BSA).

Summarizing the available literature, Table 1 describes the perioperative parameters included in the definition of RV failure.

## 3. Anatomy and Physiology of the Right Ventricle

In contrast to the systemic circulation, the pulmonary vascular system is characterized by low impedance and pressure; consequently, the RV is structured to operate optimally with a high-volume inflow and low afterload status [10,11]. The main anatomical difference with the left ventricle (LV) is the thin-wall structure, which is arranged in a triangular shape from a lateral view and in a crescent shape in a cross-sectional view. The RV has a “free wall” and shares the interventricular septum (IVS) with the LV, making it easy to understand how contractions on the right and left are influenced by each other [12]. Trabeculations within the ventricular chamber are essential for maintaining the shape of the RV during contractions and aiding in the efficient ejection of blood [13].

The tricuspid valve is formed by a fibrous anulus and three leaflets, named after their position (septal, anterior, and posterior). Ventricular papillary muscles are attached to the cusps through the chordae tendineae. Unlike the LV, the RV has just two muscular layers, one superficial and parallel oriented and one deeper and longitudinal oriented, which extends for the entire length of the ventricle. This explains the predominant longitudinal contraction of the free wall during systole from the base to the apex, “squeezing” the blood into the pulmonary system [14]. Also, the IVS plays an important role on right systole during LV contraction by maintaining a balanced intraventricular pressure and promoting efficient blood flow into pulmonary circulation [15].

Unlike the left ventricle, the right ventricle is perfused in both systole and diastole due to the lower pressure within the chamber [16]. This, together with the lower stroke works (around one-fourth/one-sixth of the LV) generated during the cardiac cycle and the presence of numerous collateral vessels make the healthy RV less susceptible to ischemia compared to the LV. On the other hand, acute and drastic changes in systemic and pulmonary pressures could have high impacts on RV perfusions [14].

Just like the LV, impairment in preload, contractility, or afterload may cause RV dysfunction, especially in the immediate postoperative period. Like any heart chamber, the RV also respects the Frank–Sterling relationship, and this is important especially in the dilated and D-shaped RV, while it is less important in the healthy RV which, thanks to its thinness, can tolerate large volumes (100–120 mL at the end of diastole) without making significant changes in pressure. In fact, thanks to its compliance, the RV replies to an increase in volume (preload) with overdistension. A progression in distension can alter the normal shape of the IVS, eliminating the IVS action in RV contractility—a phenomenon known as ventricular interdependence. Normally, ventricular interdependence plays a significant role in supporting overall RV function, emphasizing the interconnected nature of both ventricles within the heart [17].

Due to the lower pressure and higher distensibility of the pulmonary circulation, the RV wall is significantly thinner (2–4 mm) compared to the left ventricle (8–12 mm). This thinness allows the right ventricle to handle high volumes without experiencing significant changes in pressure. However, the right ventricle is highly afterload-dependent, and is primarily influenced by the pulmonary vascular resistance (PVR), pulmonary arterial pressure (PAP), and left ventricular function. Acute increases in afterload are poorly tolerated by the right ventricle, while chronic increases can be compensated through wall thickness augmentation [18]. A cardiopulmonary bypass, pulmonary reperfusion, hypoxia, hypercapnia, hypothermia, acidosis, and high tidal volumes and positive end-expiratory pressure (PEEP) during mechanical ventilation are common causes of PVR (and consequently RV afterload) augmentation after cardiac surgery [19,20].

## 4. Mechanism and Risk Factors of RV Dysfunction after Cardiac Surgery

RV dysfunction after cardiac surgery can result from several mechanisms and risk factors. The primary mechanisms involve changes in preload, afterload, and contractility. Key causes include volume overload, pressure overload, myocardial ischemia, inflammation, oxidative stress, pulmonary embolism, pericardial effusion, arrhythmias, and various mechanical factors.

There is a continuing debate regarding etiology and clinical implications of reduced perioperative RV function. Intrinsic RV myocardial dysfunction in the setting of cardiac surgery is one of the most challenging causes to prevent and to treat. Reports indicate that acute reductions in RV function can occur during the intraoperative phase following a cardiopulmonary bypass (CPB), and this reduction is independent of specific procedural characteristics and pericardiotomy [21]. Ischemic/reperfusion injury, a well-established phenomenon following a cardiopulmonary bypass, can have adverse effects on ventricular function. Prolonged ischemic time during surgery has been associated with more pronounced deleterious effects. Studies have demonstrated a correlation between an ischemic insult and severe RV dysfunction in some patients [22]. Several risk factors that can enhance the deleterious effects of ischemia and contribute to severe RV dysfunction after cardiac surgery have been identified. A shorter aortic cross-clamp time, approximately 50–70 min, and a higher baseline RV function seem to be associated with a better RV function at the end of surgery. Choice of cardioplegia, cardioplegia delivery, and CPB weaning characteristics were not associated with a clinically significant decrease in RV function [23]. However, the ischemic insult has not resulted in severe RV dysfunction post-cardiotomy in many patients [23]. One key to the successful treatment of postoperative RV failure is the early identification of patients at the highest risk, with a focus on promptly detecting perioperative dysfunction. Preliminary studies on the molecular mechanisms underlying RV dysfunction have highlighted the potential factors contributing to its development. One hypothesis suggests that the downregulation of the sarcoplasmic reticulum calcium ATPase (SERCA2) may play a role in a decrease in RV function following an ischemic insult. SERCA2 has been implicated in calcium handling and contractility of the right ventricle, making it a key candidate for further investigation into the molecular mechanisms behind post-ischemic RV dysfunction [24]. Oxidative stress, a pivotal factor in the pathophysiology of RV dysfunction, emerges as a significant player in this multifaceted condition. Under conditions of increased oxidative stress, there is an imbalance between the production of reactive oxygen species (ROS) and the ability of antioxidant defenses to neutralize them. In RV dysfunction, this oxidative imbalance can lead to cellular damage, inflammation, and impairment in the function of the right ventricle. However, further research is needed to fully understand the intricate molecular pathways involved in RV dysfunction.

## 5. Special Considerations: RV Dysfunction after LVAD

Right ventricular dysfunction following left ventricular assist device (LVAD) implantation remains a leading cause of perioperative morbidity, multi-organ dysfunction, and overall mortality [25,26,27,28,29]. Despite the well-known advantages coming with the use of the newer, centrifugal as well as axial continuous-flow LVAD devices, and the development of several clinical prediction scores, RV dysfunction incidence remains high in the early postoperative period, ranging from 24% to 40% and declining to 8% thereafter.

RV dysfunction at 1 or 3 months post-LVAD is a common and frequently transient condition, which, if resolved, is associated with a relatively favorable prognosis. Conversely, de novo late RHF post-LVAD (>6 months) is more frequently a persistent disorder, and it is associated with increased mortality [30].

The background of heart failure (HF) seems to play a significant role in the incidence of RV dysfunction after LVAD implantation. Patients with non-ischemic HF undergoing LVAD placement have been shown to be at an increased risk of early RV dysfunction compared to those with ischemic HF (increased risk by an absolute value of 5.1%). However, no differences in the occurrence of RV dysfunction between ischemic and non-ischemic etiology were observed after 2- and 4-year follow-ups, and the lack of a uniform definition of acute RV dysfunction makes the right ventricular dysfunction incidence assessment really challenging [31].

Tricuspid regurgitation (TR) is common in patients with end-stage heart failure. Pre-LVAD TR is part of an interplay with other risk factors (e.g., right ventricular dysfunction, pulmonary hypertension, and renal and/or liver dysfunction) [32]. Tricuspid annulus diameter has been recently recognized as an independent predictor of late right ventricular dysfunction after LVAD placement [26,32]. Interestingly, pre-LVAD tricuspid valve repair/replacement has been associated with an increased risk for right ventricular dysfunction incidence at follow-ups 3 months postoperatively, possibly indicating the presence of subtle, unidentified RV dysfunction in these patients [26]. The long-term effect of TR after device implantation on long-term mortality remains unknown [33].

Worsening TR commonly occurs immediately after device implantation because of an increased venous return and leftward shift of the interventricular septum. More specifically, the increased blood flow through the left pump determines an increased venous return to the right ventricle. At the same time, the left ventricular unloading determines a progressive leftward shift of the IVS. All these factors may potentially cause an acute RV dilatation, with TR occurrence or worsening of a pre-existing TR. New post-implant moderate or severe TR is a predictor of post-implant right ventricular dysfunction and poor long-term (>10 years) survival [33]. However, TR progressively decreases in the long-term post-operative period because of a reduction in pulmonary pressures and because of an RV remodeling with regression of the tricuspid valve annulus dilatation.

On one hand, concomitant surgery of the tricuspid valve may be helpful because both preoperative and postoperative TR are associated with increased mortality. On the other hand, a less aggressive strategy is warranted because, on average, the TR will resolve after LVAD implantation without any further intervention. Current guidelines advise a consideration of tricuspid valve surgery in the presence of moderate to severe TR at baseline.

Therefore, the key point seems to be an appropriate patient selection, considering the etiology of TR, the severity of RV dysfunction, and the underlying myocardial disease. Primary TR (e.g., caused by a pacemaker or an implantable cardioverter-defibrillator lead) might not reduce spontaneously after LVAD placement, whereas functional TR probably will. However, functional TR may not only be caused by tricuspid valve annular dilatation but also by valve tethering. In the case of severe tethering, tricuspid annuloplasty may not be enough to reduce TR. However, overall, TR decreases after the LVAD is implanted, regardless of pre-LVAD pulmonary hypertension or right ventricle function. Hence, in most of the patients, additional tricuspid valve surgery may be redundant. Therefore, patient selection for concomitant tricuspid valve surgery should not be based solely on the TR grade alone. There are no claimed recommendations about tricuspid valve annuloplasty in patients with LVAD. The literature mainly comprises case series and single-center observational studies. We should consider the pathophysiology of a pre-existing TR and tailor our decisions to specific patients [3,33]. Further studies are needed to tackle this clinical dilemma in the era of durable mechanical circulatory support [26]. In some studies, primary temporary right ventricular assist device (RVAD) support with tricuspid valve repair at the time of LVAD insertion has resulted in improved early right ventricular function with a consequent higher probability of weaning from the temporary RVAD [25,26,34]. In addition, a minimally invasive approach to LVAD placement may help to further stabilize the RV contractility due to the partially preserved integrity of the pericardium [34].

In a recent study of the European Registry for Patients with Mechanical Circulatory Support (EUROMACS) registry, tricuspid valve repair concomitant with an LVAD implant did not seem to be associated with better clinical outcomes. In the analysis, concomitant tricuspid repair reduced early TR significantly after LVAD implantation; however, differences in the probability of TR disappeared during the follow-up period [25]. Prior analyses of the Society of Thoracic Surgeons database and the Interagency Registry for Mechanically Assisted Circulatory Support (INTERMACS) database revealed similar results [35,36]. In addition, a positive correlative relationship has been found between mitral regurgitation (MR) and TR post-continuous-flow LVAD implantation. The contribution of upstream moderate/severe MR to downstream impairment of RV dysfunction and TR remains unclear. The lack of associated moderate/severe MR is still linked to a lower incidence of RV dysfunction and RVAD need. Some patients might benefit the most from restoring mitral valve (MV) competency with MV repair or replacement as residual MR is coupled with moderate/severe RV dysfunction [37].

## 6. Diagnostic Options

Early diagnosis of RV disfunction in cardiac surgery patients is crucial. Delaying treatment initiation could dramatically make prognosis and outcomes worse.

### 6.1. Gold Standard: cMRI and Thermodilution Technique

At the present time, two techniques are indicated as gold standards: cardiac magnetic resonance imaging (cMRI) and hemodynamic assessment with thermodilution [38,39]. cMRI allows for non-invasive measurement of end-diastolic and end-systolic volumes with great precision, along with derived measures such as ejection fraction (EF) and stroke volume (SV). It also provides a detailed visualization of complex RV anatomy and allows the assessment of flow velocities, patterns, and valvular function using phase-contrast modalities. However, cMRI is limited in the postoperative cardiac setting due to its cost, time requirements, challenges in critically ill patients, and limited accessibility [40,41,42].

The thermodilution technique is an invasive method performed using a pulmonary artery catheter (PAC or Swan–Ganz catheter) [43]. Different studies in the past compared PAC with echocardiography, demonstrating high concordance between techniques, except in patients with high heart frequency and atrial fibrillation [44,45,46]. Typically, an increase in central venous pressure (CVP) coupled with a decrease in cardiac output (CO) is indicative of RV dysfunction to some degree. Elevation in pulmonary artery pressure (PAP) and pulmonary vascular resistance (PVR) and consequently pulmonary hypertension can be easily detected and monitored, allowing continuous adjustments in treatment. However, due to the invasiveness and complexity of PAC together with the wide spread of transesophageal echocardiography, thermodilution has lost popularity in recent years, also considering the fact that it may not detect combined RV and left ventricular (LV) impairment.

### 6.2. The Role of the Echocardiography: TAPSE, RVEF, RVFAC, TDI, Strain, 3D Assessment

Echocardiography is the most attractive and probably most used technique for evaluating RV function in the perioperative period due to its availability, reproducibility, non-invasiveness, and low-cost [47]. Thoweed et al., in their recent study on patients who underwent valvular surgery, stressed the need for a comprehensive and extended echography study, both pre- and postoperatively, to predict severe short-term outcomes [48]. Transthoracic echocardiography (TTE) is commonly used but has limitations in evaluating the RV due to its trabeculated anatomy and retrosternal position. Additionally, the surgical opening of the chest and the presence of residual air in the thoracic cavity often result in a poor acoustic window after surgery [49].

Transesophageal echocardiography (TEE) provides better resolution and visualization of the right side of the heart, including the tricuspid valve, but may have difficulties with Doppler alignment [18]; TEE is typically used intraoperatively, while the patient is under general anesthesia and is intubated.

Various echocardiographic measurements can be performed to evaluate perioperative RV function, and some of them have been correlated with postoperative course and mortality [39,50,51]. At a first glance, a thickness greater than >5 mm indicates RV hypertrophy. Moreover, RV dilatation and volume augmentation over 2/3 of the LV can be observed during RV dysfunction. Tricuspid annular plane systolic excursion (TAPSE) is a classic indicator of longitudinal RV contraction. It is the excursion of the tricuspid annulus between end-diastole and end-systole along a longitudinal plane. TAPSE is easily measured in M-mode in a 4-chamber view by placing the cursor on the free wall of the tricuspid annulus. The normal range for TAPSE is 20.7 mm ± 11.1 (SD) for males and 20.3 mm ± 8.6 (SD) for females, with values under 17 mm usually indicating some degree of dysfunction [52]. TAPSE has gained popularity thanks to its easy measurement and reproducibility [6,53,54]; however, it has some disadvantage, mainly because it does not detect regional dysfunction. Moreover, it is angle-dependent, load-dependent, and may be increased in the case of tricuspid regurgitation. A reduction in TAPSE after cardiac surgery has been confirmed in different [55,56] studies, and it is an indicator of a significant reduction in RV function after a cardiopulmonary bypass (CPB). TAPSE probably decreases because of right myocardial stunning (eg. insufficient myocardial protection), opening of the pericardium, and a change in RV contraction, which lead to a reduction in the longitudinal contraction and improvement of transverse shortening of the heart, as showed in the study of Korshin and colleagues [57].

Recent studies on the RV diastolic function (RVDD) and its predictive value for postoperative outcomes in cardiac surgery underline the prognostic value of a comprehensive assessment of the function of RV. In particular, all the authors encourage the preoperative indicators of RV filling for a more accurate assessment of the prognosis [58,59,60]. Sumin et al. [58] showed that RVDD was associated with the development of MACE within 18 months after CABG in the preoperative period, and the ratio of Et/e’t was one of the independent predictors of MACE in a multiple regression analysis, in addition to the clinical parameters (the presence of peripheral atherosclerosis and no history of PCI). Interestingly, RV diastolic dysfunction seems to be associated with short- and medium-term prognosis after CABG, and occurs in patients without initial left ventricular systolic dysfunction. Similarly, the same group [60] showed that the development of RVDD can occur without RV systolic dysfunction in patients with CAD without pronounced valvular disease and decreased left ventricular pumping function, potentially increasing the number of patients in whom an assessment of RVD can be used for prognostic purposes.

According to Kossaify et al. [61], the right ventricular ejection fraction (RVEF) is considered normal at a value of 44% or more. However, the complex shape and geometry of the RV make it challenging to obtain a precise measure of its EF with 2D echocardiography, especially when compared to left ventricular EF. A good surrogate of RVEF is the right ventricular fractional area change (RVFAC), which is the fractional difference of the area of the RV between end-diastole and end-systole; it is calculated by tracing the RV endocardium manually in a four chambers view using 2D echocardiography. An RVFAC < 35% is considered pathological [18,62]. Anyway, it must be noted that RVFAC provides a two-dimensional assessment of RV function and is reliant on manual tracing of the RV endocardium. This means it may not capture the complexities of the shape and motion of the RV accurately. Furthermore, RVFAC is less sensitive to subtle changes in RV function, and its threshold for pathology may not be as precise as more precise imaging techniques and measurements.

Tissue Doppler imaging (DTI) uses myocardial Doppler signals to assess myocardial function and contraction. The longitudinal velocity called S’ is the highest velocity of the tricuspid annulus during systole. A value lower than 9.5 cm/s is indicative of RV dysfunction. As TAPSE, it is a measure of longitudinal contractility, and it must be used carefully after cardiac surgery, when it has often been found reduced. According to Nguyen et al., an explanation for a diminished longitudinal contraction could be found in septal damage during a surgical procedure and CBP [63].

Strain is a parameter that can be determined by tracking spots on the sonograms of different cardiac circles in DTI or speckle tracking modes. In their study on 70 patients who underwent tricuspid surgery, Torres and colleagues showed that free wall longitudinal strain can be a better parameter to assess RV function in the first period after surgery, especially when compared to TAPSE and S’ [64]. In a similar way, Orde et al. have also found a significant RV strain reduction after open mitral valve surgery [65]. This technique has various limits and requires several cardiac cycle measurements with good-quality images, which are not always easy to obtain after cardiac surgery.

Finally, three-dimensional (3D) echocardiography is a recent modality that sums up different 2D images in a 3D representation [39]. It can allow more a precise study of RV anatomy and function but, more than ever, a good quality window is needed. Moreover, rhythm abnormalities, abnormal septal motion, load dependence, complexity of measurement, and poor software availability are the main limitations.

### 6.3. MUGA, Conductance Catheter Techniques/PV Loops, Nuclear Imaging/PET, Biomarkers

Several other techniques have been developed to study and monitor RV function [18]; however, their use in the perioperative setting is limited. Multi-gated acquisition (MUGA) is a non-invasive imaging method that employs radio-labeled red blood cells with a radioactive isotope to precisely delineate the internal borders of the right ventricle. By capturing multiple images during the cardiac cycle and using specialized software, MUGA provides an accurate quantification of the right ventricular ejection fraction (RVEF), a critical measure of cardiac function [66].

The conductance catheter technique is an invasive and advanced method used for comprehensive real-time assessment of RV function. It involves the insertion of a specially designed catheter equipped with electrodes into the RV and provides real-time quantification of the intraventricular volume and pressure loops. To perform measurements, a saline solution is injected into the RV through the catheter. The conductance of the surrounding tissue changes as the saline bolus enters the RV and mixes with the blood, altering the electrical properties in the catheter’s field. By continuously recording these changes in electrical conductance, the catheter provides accurate data on the RV volume and pressure variations throughout the cardiac cycle [67].

Nuclear imaging has also been used to indagate RV parameters. Positron emission tomography (PET) use, for example, has been indagated since RV muscular cells increase glucose uptake in the case of heart failure [68]. Anyway, it is worth noting that metrics such as the ejection fraction (EF) do not consistently align with those derived from the gold standard cardiac magnetic resonance imaging (cMRI). This discrepancy can arise due to there being differences in imaging modalities, accuracy in delineating RV borders, and the sensitivity of each technique to subtle changes in RV function.

Finally, as discussed in the work of Jabagi et al., few studies have examined different biomarkers for RV dysfunction detection, such as ST2/sST2, CRP, Galectin3 cTN/hscTn, and the more famous BNP/NT-proBNP [69]; however, validation is warranted before clinical application.

## 7. The Clinical Approach

Perioperative diagnosis and monitoring of RV failure and dysfunction cannot rely on complicated imaging techniques or extremely advanced cardiography calculations; however, it must be fast and easily accessible for the anesthetist in the operating room and for the intensivist in the ICU. In their review, Varma and colleagues provided a simplified approach to identify key findings of RV dysfunction based on (1) echo signs of RV dilation; (2) reduction of TAPSE; and (3) plethoric inferior vena cava as a sign of elevated preload [18].

In our experience, RV dysfunction after cardiac surgery should be suspected starting from the intraoperative period, especially after weaning from the cardiopulmonary bypass (CBP) (Table 2). Direct visualization or TEE examination of the RV, when available, and visual reduction of contractility in comparison with the pre-CBP period must be noted and reported to the ICU physicians.

Moreover, some preoperative risk factors and alarm signs, such as pulmonary hypertension or tricuspid regurgitation, should be seriously taken into account. In the case of serious pulmonary hypertension or preoperative RV dysfunction, we suggest placing a pulmonary catheter before starting the intervention to better monitor hemodynamic parameters during and after surgery.

In the ICU, RV dysfunction detection should be based on echo signs as well on hemodynamic signs. RV dilation indexes and a reduction in TAPSE should be used to evaluate contraction and function. If a pulmonary catheter has been placed, an augmentation in PVC with a reduction in CO is a strong indicator of RV dysfunction. In the absence of a pulmonary catheter, an augmentation of PVC detected with a central venous catheter associated with impairment of other hemodynamic parameters, such as arterial invasive pressure, could be an early alarm sign.

By integrating these practical approaches, clinicians can promptly identify and monitor RV failure and dysfunction in the perioperative period, allowing for timely interventions and optimizations of patients’ care.

## 8. Management of RV Failure after Cardiac Surgery

One of the milestones of RV dysfunction management is early detection and treatment initiation. Therapy should be focused on optimizing preload, contractility, perfusion, and afterload of the right ventricle. Different strategies could be adopted, and should always be tailored to the patient and to the specific echocardiography and hemodynamic information which is constantly evolving [18,62,70]. The data derived from echo and hemodynamic monitoring can be used to understand the efficacy of the treatment and to adjust it through the phases of the postoperative period.

### 8.1. Intravenous Drugs

Intravenous drugs can be used to optimize different actors which play fundamental roles in the normal function of the RV. Inotropes are used to improve contractility, both in LV and RV impairment. Dobutamine is a classic beta agonist drug which acts by increasing the contractility of the muscular cells of the heart; moreover, it causes vasodilation, decreasing pulmonary and systemic vascular resistance [71]. However, if dosage is increased up to 10 mcg/kg/min, tachycardia and augmentation of oxygen consumption is very likely.

Milrinone and enoximone, two selective phosphodiesterase III inhibitors, are other inotropic drugs which also cause a decrease in PVR, playing a role both on contractility and afterload [72]. Their use is crucial in RV dysfunction treatment because they have a strong inotropic effect and they do not increase heart frequency and oxygen consumption, making them an important alternative, especially when an ischemic condition is present [73]. They are usually paired with a vasopressor drug because they often cause systemic pressure to decrease due to vasodilation.

Levosimendan, an inotropic drug which is diffusely used for pre-conditioning impaired ventricular function before surgery, improves contractility by increasing the sensitivity of troponin C to calcium inside muscular cells [74]. Like phosphodiesterase III inhibitors, levosimendan does not increase oxygen consumption and hearth rate; moreover, it causes an opening of the adenosine triphosphate-dependent potassium channels and consequent relaxation of smooth muscular cells and vasodilation. Currently, milrinone, enoximone, and levosimendan are the most used inotropes in RV dysfunction.

Pulmonary vasodilators, on the other hand, are used to reduce RV afterload. They can be used alone or, more frequently, paired with an inotropic drug. As a side effect, intravenous vasodilators also usually reduce systemic arterial pressure.

Phosphodiesterase 5 inhibitors, such as sildenafil, are the most used; these inhibitors block the degradation of cyclic guanosine monophosphate, finally reducing PVR and SVR. Sometimes they can be used along with a inhaling pulmonary vasodilator or, most commonly, in the preoperative period or when the patient is weaned from mechanic ventilation and inhaled drugs are stopped [75].

Other intravenous vasodilators which are less commonly used in clinical practice include prostacyclin and endothelin receptor antagonists. Endothelin receptor antagonists work by blocking the endothelin receptors which are in the smooth muscles cells in the vasal system, leading to a reduction in pulmonary pressure; however, their hepatotoxicity limits their clinical use [76].

### 8.2. Inhalator Drugs

Inhaled pulmonary vasodilators are commonly used in RV dysfunction after cardiac surgery, especially in the operating room after weaning from the CPB or in the first period in an ICU, when patient is still intubated.

In clinical practice, inhaled nitric oxide (iNO) is the most used [1]. It is potent and has a rapid onset and short half-life; moreover, its action is selective on pulmonary circulation, leading to a decrease in PVR thanks to the augmented releasing of cyclic guanosine monophosphate in pulmonary vasal cells. Inactivation with hemoglobin in the micro-circulation of lungs provides an example of its selective action. It must be noted that rapid discontinuation of its continuous administration can cause rebound pulmonary hypertension [77].

Prostacyclin is a strong pulmonary vasodilator; however, it usually provides important systemic hypotension when administered intravenously, and its inhaled form can bypass this important side effect. It is widely used in the setting of augmented PVR after cardiac surgery [78].

### 8.3. Ventilatory Strategies

Ventilatory parameters play an important role in RV preload and afterload optimization. In fact, mechanical ventilation increases pressure inside the thorax during inspiration, increasing RAP and RV afterload. The use of augmented tidal volumes could worsen pulmonary hypertension, decreasing RV ejection and stroke volume, especially when the RV is already dilated and dysfunctional. On the other hand, hypoxia, hypercapnia, and acidosis should be avoided to avoid the well-known phenomenon called hypoxic pulmonary vasoconstriction, which is exclusive to pulmonary circulation. Therefore, small lung volumes can also increase PVR and worsen RV afterload and function. Finally, high PEEP levels can clench pulmonary capillaries in well-ventilated lungs areas, increasing ventilation/perfusion mismatch and hypoxia [18,62].

### 8.4. Volumes Strategies

Fluid management is essential to optimize the circulating volume; in fact, both hypovolemia and hypervolemia could have a negative impact on CO during RV dysfunction. In case of volume depletion, management should target reaching a RV filling pressure of 8–12 mmHg with volume reintegration [79]. However, volume overload, especially in the setting of an over-distended RV, should be avoided more than ever [80]. With excessive distension, shifting of the interventricular septum to the left side could also decrease LV diastolic compliance and finally decrease LV CO. In the case of a high preload, fluid removal should be implemented with intravenous diuretics, such as furosemide, or by starting CRRT when necessary.

### 8.5. Mechanical Support

When RV dysfunction does not respond to maximal medical therapy, mechanical support of the right ventricle should be evaluated [70].

Although its role in RV dysfunction is controversial, the intra-aortic balloon pump (IABP) could be used to improve diastolic pressure and coronary perfusion and to reduce afterload, providing support to a post-cardiac surgery dysfunctional RV [81].

Right ventricular assistant devices (RVAD) can be implanted surgically or percutaneously with central or peripheral cannulation. Usually, they provide support throughout extracorporeal devices. RVAD can be used as a bridge to recovery, especially in patients with ischemic RV dysfunction [82]. Typical configurations comprehend continuous flow with right atrial and pulmonary artery cannulations.

When RV dysfunction occurs due to an augmented PVR, venous/arterial ECMO is the best mechanical support option. VA-ECMO can decrease PAP and loosen up RV, maintaining the output of the RV. VA-ECMO can have a negative impact on LV CO, leading to LV failure and an increase in PVR. When PVR increases irreversibly and weaning from mechanical support becomes problematic, VA-ECMO could be considered as a means to perform a transplant [83].

## 9. Conclusions

RV biomechanics have been shown to be crucial for patients undergoing cardiac surgery, and the interplay between the RV and other apparatus, such as pulmonary vasculature, or the left ventricle is of utmost importance to tackle different loading conditions to meet flow demands. At present, the definition of RV dysfunction remains debated, but the precise mechanism leading to RV failure (pressure overload, volume overload, or intrinsic myocardial process) should be clearly defined to provide prompt support and an adequate treatment (unloading vs. inotropes). The implementation of diagnostic tools and the use of novel RV-targeted therapies seem to reduce the degree of dysfunction. However, research gaps should be overcome in the near future to smoothen the detrimental clinical outcome associated with RV dysfunction.

**Table 1:** Perioperative parameters included in the definition of “right ventricular failure”.

Parameter	Supporting Literature
**RVFAC reduction (<35%)**	Maslow et al. [84]; Dávila-Román et al. [85]; Reichert et al. [86]; Denault et al. [87]; Haddad et al. [88].
**CVP augmentation (>18 mmHg)**	Kaul et al. [89]; Schuuring et al. [90].
**CI reduction (<2.2, with normal LAP)**	Kaul et al. [89].
**RV dilation (RVESV > 3 mm)**	Dávila-Román et al. [85]; Levy et al. [1].
**Need for RVAD**	Moazami et al. [91]; Gudejko et al. [92]; Ochiai et al. [93]; Grant et al. [94]; Kavarana et al. [95]; Matthews et al. [96]; Drakos et al. [97]; Kormos et al. [27]; Fitzpatrick et al. [98]; LaRue et al. [99].
**Hemodynamic instability**	Denault et al. [87]; Levy et al. [1].
**RVMPI augmentation (>0.5)**	Haddad et al. [88].
**Impaired RV wall motion (echo visualization)**	Denault et al. [87]; Dávila-Román et al. [85]; Levy et al. [1].
**TAPSE reduction (<16 mm)**	Schuuring et al. [90]; Levy et al. [1].
**RV S’ reduction (<11 cm/s)**	Schuuring et al. [90]; Levy et al. [1].
**RV longitudinal strain augmentation (>21%)**	Ternacle et al. [100].
**Inotropic support (for >14 days)**	Gudejko et al. [92]; Grant et al. [94]; Kavarana et al. [95]; Matthews et al. [96]; Drakos et al. [97]; Kormos et al. [27]; LaRue et al. [99].
**Pulmonary vasodilator support (for >48 h)**	Gudejko et al. [92]; Matthews et al. [96]; Drakos et al. [97]; Levy et al. [1].

RVFAC = right ventricular fractional area change; CVP = central venous pressure; CI = cardiac index; RVAD = right ventricular assist device; RVMPI = right ventricular myocardial performance index; TAPSE = tricuspid annular plane systolic excursion; S’ = longitudinal velocity; LAP = left atrial pressure; RVESV = right ventricular end-systolic volume.

**Table 2:** Mechanisms potentially leading to right ventricular dysfunction after cardiac surgery.

Common procedures (right ventricular dysfunction related to a cardiac surgery per se or a cardiopulmonary bypass)	Conditions at increased risk	Preoperative right ventricular dysfunction Tricuspid valve surgery Pulmonary hypertension (primary or secondary)
	Cardiopulmonary bypass Myocardial protection	Right atrial cannulation Pericardiectomy and alteration in diastolic function Myocardial stunning Myocardial mal-protection (especially if chronic coronary occlusion is present) Coronary air embolism (especially in valvular procedures) Inadequate right ventricular unloading
	Operative management	Protamine or vasopressors => increased pulmonary vascular resistance Lung deflation => increased pulmonary arterial pressure Reperfusion injury => increased pulmonary arterial pressure
Specific procedures (right ventricular dysfunction related to specific cardiac procedures)	Heart transplantation	Autonomic storm => decreased systemic vascular resistance Ischemic time after procurement Cardioplegic solution and preservation solution Perioperative mechanical circulatory support
	Pulmonary endarterectomy	Preoperative right ventricular dysfunction Reperfusion injury => increased pulmonary arterial pressure
	Left ventricular assist device implantation	Increased right ventricular stress due to left ventricular unloading Left ventricular/right ventricular uncoupling Splanchnic congestion due to an increased venous return Alterations in the septal and anterior wall
